# Evaluation of the role of B7-H3 haplotype in association with impaired B7-H3 expression and protection against type 1 diabetes in Chinese Han population

**DOI:** 10.1186/s12902-020-00592-7

**Published:** 2020-08-12

**Authors:** Sisi Ding, Yimei Shan, Lili Sun, Sicheng Li, Rong Jiang, Xin Chang, Ziyi Huang, Jing Sun, Cuiping Liu, Chen Fang, Xueguang Zhang

**Affiliations:** 1 Jiangsu Institute of Clinical Immunology & Jiangsu Key Laboratory of Clinical Immunology, First Affiliated Hospital of Soochow University, Suzhou, 215007 Jiangsu People’s Republic of China; 2 Department of Endocrinology, Second Affiliated Hospital of Soochow University, Suzhou, 215004 Jiangsu People’s Republic of China; 3 Institute of Medical Biotechnology, Suzhou Health College, Suzhou, 215009 Jiangsu People’s Republic of China

**Keywords:** Type 1 diabetes, SNP, mB7-H3, sB7-H3

## Abstract

**Background:**

Type 1 Diabetes (T1D) is a T cell-mediated autoimmune disorder caused by the destruction of insulin-secreting cells. B7-H3 (CD276) plays a vital role in T cell response. However, B7-H3 expression and its clinical significance in T1D remain unclear. The aim of this study was to investigate the correlations between the expression of B7-H3 and clinical parameters in T1D patients. The possible role of B7-H3 gene variants with T1D was also discussed.

**Methods:**

Four B7-H3 single nucleotide polymorphisms (SNPs) were genotyped in 121 T1D patients and 120 healthy controls by polymerase chain reaction (PCR) direct sequencing. Expression of membrane B7-H3 (mB7-H3) in peripheral blood lymphocytes was determined by flow cytometry. Levels of soluble B7-H3 (sB7-H3) in serum were analyzed by enzyme linked immunosorbent assay (ELISA).

**Results:**

The B7-H3 haplotype T-A-C-T was less frequently observed in T1D patients compared to the controls (OR: 0.31, 95% CI: 0.16–0.61). B7-H3 expression on monocytes showed significant upregulation in T1D patients and was positively correlated with several clinical features including ALT, fast C-peptide 120 min, HbAlc, IFN-γ, IL-6 and TNF-α (*P* < 0.05). The concentration of sB7-H3 in serum increased in T1D patients *(P* < 0.0001). We also observed that B7-H3-T-A-C-T was associated with the decreased release of sB7-H3 but not the membrane form.

**Conclusions:**

B7-H3 may act as a potential biomarker related to the pathogenesis of T1D. The B7-H3-T-A-C-T polymorphism variant is associated with the low risk of T1D as well as less release of sB7-H3.

## Background

Type 1 diabetes (T1D) is a chronic autoimmune disease that leads to the selective loss of insulin-producing β-cells by activating the T cells [1]. The co-stimulatory and co-inhibitory pathways modulate T cells activation and hence play a vital role in T1D. Scientists had made several advances in the past to understand how co-stimulatory and co-inhibitory pathways affect T1D, such as CD28/B7.1, CD40/CD40L, ICOS/ICOSL and PD1/PD-L1 [2–6]. B7-H3, as a co-stimulatory molecule, which belongs to the B7 immunoglobulin superfamily, is frequently increased in response to the autoantigens and pathogens during host T cell immunoregulation [7].

The biological effect of B7-H3 for its co-stimulatory and co-inhibitory properties remains controversial [8, 9]. There are studies revealing the capability of B7-H3 in promoting CD4^+^ and CD8^+^ T cell proliferation, cytotoxic T lymphocytes (CTLs) induction and interferon-gamma (IFN-γ) secretion in vitro [8, 10]. However, some studies speculated that B7-H3 may also have the inhibitory function. B7-H3 could inhibit the proliferation of CD4^+^ and CD8^+^ T cells and crucial transcription factors namely nuclear factor of activated T-cells (NFAT), nuclear factor kappa B (NF-κB) and activator protein 1 (AP-1) [10–12]. In neuroblastoma, B7-H3 exerted a protective role and thereby downregulated natural killer (NK) cell function [13]. Additionally, it was reported that the soluble and membrane form of B7-H3 could inhibit NK cell-mediated lysis in glioma [14]. Recently, we demonstrated the upregulated expression of sB7-H3 in T1D patients and found the correlation with gender as well as serum levels of creatinine (Cr), blood urea nitrogen (BUN), albumin to creatinine ratio (ACR) and high-density lipoprotein (HDL) [15]. Nevertheless, the expression and function of B7-H3 in T1D remains unknown.

In the present study, we analyzed the expression of B7-H3 and its clinical significance in T1D patients. We aimed to investigate four single nucleotide polymorphisms (SNPs) of B7-H3 genes to determine its associations with T1D risk in order to find the correlations among B7-H3 haplotype, secretion of soluble B7-H3 and biochemical parameters in T1D.

## Methods

### Patients and samples

In this study, we included the blood samples of 121 patients affected by T1D during the period 2015 and 2018 at the Second Affiliated Hospital of Soochow University. ‘The T1D Exchange Clinic Registry’ was chosen as the diagnostic criteria for T1D [16]. We matched 120 healthy controls with the T1D patients based on their age, gender and race, and they did not suffer from conditions like infectious diseases, cancer, acute or chronic inflammatory diseases. From the selected patients, we enrolled 52 T1D patients and 55 healthy controls to detect the expression of membrane form of B7-H3 on monocytes, and 103 T1D patients and 73 healthy controls to examine the concentration of soluble B7-H3. We obtained approval for this study from the Ethics Commitee of the Second Affiliated Hospital of Soochow University.

### DNA extraction and polymorphism genotyping

We followed the standard procedure to isolate the genomic DNA from peripheral blood leucocytes and stored the DNA samples at −20 °C. We selected four SNPs in the B7-H3 gene based on the report presented by our team in the past [17]. Primers were designed according to the gene sequence (accession number AF363458) in Genbank to amplify the B7-H3 fragments. B7-H3 mutations were screened by direct sequencing the PCR products. For 20 samples, PCR amplification and sequencing of genomic DNA were repeated up to 2 times to confirm complete concordance.

### Flow cytometry

We analyzed the serum and plasma using the routine procedures at the Jiangsu Institute of Clinical Immunology. The expression of membrane B7-H3 on monocytes in peripheral blood from patients and controls was detected by flow cytometry as previously described [18]. The gating strategy for the CD14^+^B7-H3^+^ cell subset was described in Supplementary Fig. 1, Additional File 1. FlowJo software (Tree Star, Ashland, OR) was used to analyze the data.

### Soluble B7-H3 measurement

Serum collected from the peripheral blood of the T1D patients and healthy controls were stored at −80 °C. We used the lab-developed ELISA kit to detect the levels of sB7-H3 [19]. The microplate reader (Bio-Rad Laboratories, Hercules, CA, USA) was used to analyze the OD value at 450 nm.

### Cytokines production

Cytometric bead array system (CBA) (BD-Pharmingen, CA) was used to quantify the cytokines including IL-2, IL-4, IL-6, IL-10, IL-17A, IFN-γ and tumor necrotic factor-alpha (TNF-α). 50 μl sera were mixed with 50 μl beads in each tube to observe the reaction seperately. PE detection reagent was then added and incubated for 3 h at room temperature. Finally, flow cytometry and BDTM CBA Software were applied to get the acquired data.

### Statistical analysis

The statistical analysis was made using SNPstats software (http://bioinfo.iconcologia.net/SNPstats) [20]. Similar to logistic regression (LR), the odds ratio (OR) and 95% confidence interval (CI) were revealed. The magnitude of linkage disequilibrium (LD) was evaluated using the D’ and r^2^ calculations. Statistical analysis was performed by SPSS 22.0 (IBM Corporation, USA). Student’s t test was included and the differences between the groups were analyzed. Spearman’s rank correlation analysis was used and the correlation between B7-H3 protein levels and clinical variables were analyzed. *P* < 0.05 was considered statistically significant. Finally, we used GraphPad Prism5.0 (Graph-Pad Software, La Jolla, CA) for drawing graphs.

## Results

### Single nucleotide polymorphism (SNP) analysis

Patients’ clinical characteristics were summarized in Table 1. We successfully genotyped all the four SNPs in 121 T1D patients and 120 healthy controls (See Supplementary Fig. 2, Additional File 2). Table 2 showed the allele and genotype distribution of these four SNPs. The genotypic distribution in controls for all four SNPs was conformed to Hardy-Weinberg equilibrium (HWE). However, the genotype and allele distributions of all the four SNPs differed significantly between the T1D patients and the healthy controls (*P* < 0.05). The dominant model showed the smallest Akaike’s information criterion value (See Supplementary Table 1, Additional File 3). Thus, it was enrolled as the best inheritance model.

**Table 1:** Clinical features of study population

	T1D	HC	*P* value^*^
Sample size, n	121	120	
Age, years	28.5 (8–53)	29.4 (24–49)	0.75
Gender, female(n%)	71 (58.68%)	72 (60.00%)	0.83
Duration, years	7 (0–39)	–	
Fasting venous blood glucose (mmol/L)	11.9 (3.99–22.31)	–	
Cr (umol/L)	56.19 (26.00–314.00)	–	
BUN (umol/L)	4.43 (1.40–21.50)	–	
UA (umol/L)	275.68 (81.00–540.00)	–	
ALT (IU/L)	28.90 (1.00–414.00)	–	
AST (IU/L)	21.32 (6.00–137.00)	–	
TC (mmol/L)	4.42 (0.81–10.91)	–	
TG (mmol/L)	1.09 (0.21–5.97)	–	
LDL (mmol/L)	2.63 (1.10–7.37)	–	
HDL (mmol/L)	1.52 (0.71–2.82)	–	
HbA1c (%)	9.07% (5.30–18.4%)	–	
Fasting C-peptide 0 min (ng/ml)	1.62(< 0.01–35.00)		
Fasting C-peptide 120 min (ng/ml)	0.59(< 0.01–2.27)	–	
Anti-ICA positive(%)^#^	18 (28.57%)	–	
Anti-GAD positive(%)^#^	25 (39.68%)	–	
Anti-IAA positive(%)^#^	7 (11.11%)	–	
Ketosis	51 (42.15%)	–	
IFN-γ (pg/ml)	3.07 (0–19.30)	–	
IL-6(pg/ml)	4.16 (0–15.63)	–	
TNF-α (pg/ml)	1.46 (0–17.23)	–	

Cr for creatinine, BUN for blood urea nitrogen, UA for uric acid, ALT for alanine aminotransferase, AST for aspartate transferase, TC for cholesterol, TG for triglycerides, LDL for low-density lipoprotein, HDL for high-density lipoprotein, ICA for islet cell antibodies, GAD for glutamic acid decarboxylase antibody, IAA for insulin autoantibody. ^*^P value is based on the statistical analysis by the Mann-Whitney U test or the χ2 test assessing overall group differences. ^#^Not all the patients have the antibody results, only 63 patients received antibody screening

**Table 2:** Four markers of the B7-H3 gene investigated in Han Chinese patients with T1D disease

	dbSNP	chr position and	Frequency(%)				Frequency(%)			HWE
		mRNA position	Allele			*P*	Genotype			*P*
SNP1	rs7173448	73,994,806/524		C	T		C/C	C/T	T/T	
		C → T	Cases	0.91	0.09		0.85	0.13	0.02	0.16
			Controls	0.82	0.18	0.0064	0.66	0.31	0.03	
SNP2	rs7173476	3,994,847/565		C	A		C/C	C/A	A/A	
		C → A	Cases	0.91	0.09		0.85	0.13	0.02	0.16
			Controls	0.82	0.18	0.0064	0.66	0.31	0.03	
SNP3	–	73,996,569/1359		A	C		A/A	A/C	C/C	
		A → C	Cases	0.91	0.09		0.85	0.13	0.02	0.16
			Controls	0.82	0.18	0.0064	0.66	0.31	0.03	
SNP4	rs145827704	73,996,577/1367		C	T		C/C	C/T	T/T	
		C → T	Cases	0.91	0.09		0.85	0.13	0.02	0.16
			Controls	0.82	0.18	0.0064	0.66	0.31	0.03	

Traits were adjusted for age and sex in the additive genetic model

### Linkage disequilibrium (LD) and haplotypes association analysis

We calculated the pairwise LD between the four SNPs of the B7-H3 for both T1D patients and healthy controls in Chinese Han population. We detected strong LD (D’ > 0.97) between some pairs of the markers in the B7-H3 (See Supplementary Fig. 3, Additional File 4). We found that the association analysis of the haplotypes with T1D and the genotypes by LR remained similar (Table 3). Moreover, our results demonstrated the significant correlation between haplotype T-A-C-T and the disease (*P* < 0.0001); T-A-C-T haplotype was less frequently observed in cases when compared to the controls (OR: 0.31, 95% CI: 0.16–0.61). These results indicated that haplotypes might act as a protective phenotype in Chinese Han patients with T1D. We further adjusted the association analysis of the haplotypes by sex.

**Table 3:** Estimated haplotype frequencies and the association analysis with T1D

Haplotype	Sequence	Cases	controls	Total	OR(95% CI)	P value
1	CCAC	0.9	0.8178	0.8585	1	
2	TACT	0.081	0.1822	0.1321	0.31 (0.16–0.61)	8e-04

Global haplotype association *p*-value: < 0.0001

Traits were adjusted for age and sex in the additive genetic model

### Increased membrane B7-H3 expression in T1D and its correlation with disease activity

**Fig. 1:**
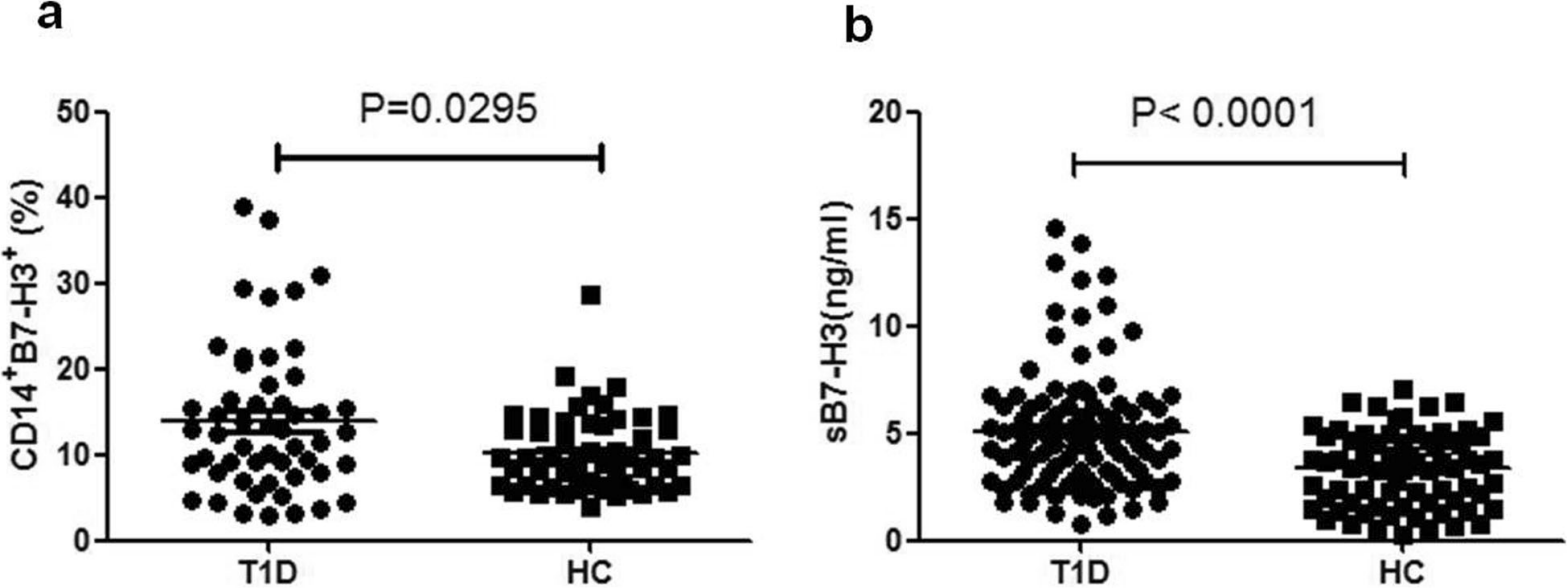
Expression of B7-H3 in HC and T1D patients. **a**. Increased expression level of mB7-H3 on CD14^+^ monocytes in PB samples from T1D patients (*n* = 52) when compared with that from controls (*n* = 55, *P* < 0.05). **b**. The concentration of sB7-H3 is increased in T1D patients (*n* = 103) when compared with controls (*n* = 73, *P* < 0.0001)

Of the 52 T1D patients and 55 healthy controls enrolled for flow cytometry analysis, the expression of B7-H3 on CD14^+^ monocytes was significantly higher in T1D patients (13.913 ± 8.640 vs 10.142 ± 4.596, *P* = 0.0295) (Fig. 1a). The mean fluorescence intensity (MFI) of mB7-H3 on CD14^+^ monocytes in PB samples from HC and T1D were also analyzed (See Supplementary Fig. 4, Additional File 5). Details about the statistical analysis of the correlation between clinical features and membrane levels of B7-H3 of T1D patients were summarized in Table 4. Our results revealed that B7-H3 had positive correlation with alanine aminotransferase (ALT) (*r* = 0.4135, *P* = 0.0080), fast C-peptide 120 min (*r* = 0.5515, *P* = 0.0117), hemoglobin A1c (HbAlc) (*r* = 0.3935, *P* = 0.0210), IFN-γ (*r* = 0.5943, *P* = 0.0003), IL-6 (*r* = 0.4057, *P* = 0.0212) and TNF-α (*r* = 0.5482, *P* = 0.0012). But the remaining parameters were not statistically significant.

**Table 4:** The correlation of clinical features and membrane levels of B7-H3

	R	p
Age, years	0.1390	0.3681
Duration, years	−0.1787	0.3044
Random blood glucose (mmol/L)	0.05984	0.4600
Cr (umol/L)	−0.02359	0.8821
BUN (umol/L)	0.1225	0.4397
UA (umol/L)	−0.05984	0.7066
ALT (IU/L)	0.4135	0.0080
AST (IU/L)	0.2989	0.0610
TC (mmol/L)	−0.1614	0.3133
TG (mmol/L)	0.1638	0.3062
LDL (mmol/L)	−0.1944	0.2358
HDL (mmol/L)	−0.1763	0.2829
Anti-ICA positive(%)	−0.2444	0.1449
Anti-GAD positive(%)	−0.1639	0.3323
Anti-IAA positive(%)	−0.2125	0.2067
Ketosis	0.3924	0.0870
GGT	0.3167	0.1075
Fasting C-peptide 0 min (ng/ml)	0.0907	0.5679
Fasting C-peptide 120 min (ng/ml)	0.5515	0.0117
HbA1c(%, mmol/mol)	0.3935	0.0210
IFN-γ (pg/ml)	0.5943	0.0003
IL-6(pg/ml)	0.4057	0.0212
TNF-α (pg/ml)	0.5482	0.0012

Cr for creatinine, BUN for blood urea nitrogen, UA for uric acid, ALT for alanine aminotransferase, AST for aspartate transferase, TC for cholesterol, TG for triglycerides, LDL for low-density lipoprotein, HDL for high-density lipoprotein, ICA for islet cell antibodies, GAD for glutamic acid decarboxylase antibody, IAA for insulin autoantibody

### Association between B7-H3 haplotypes and the expression of B7-H3 protein in T1D patients

**Fig. 2:**
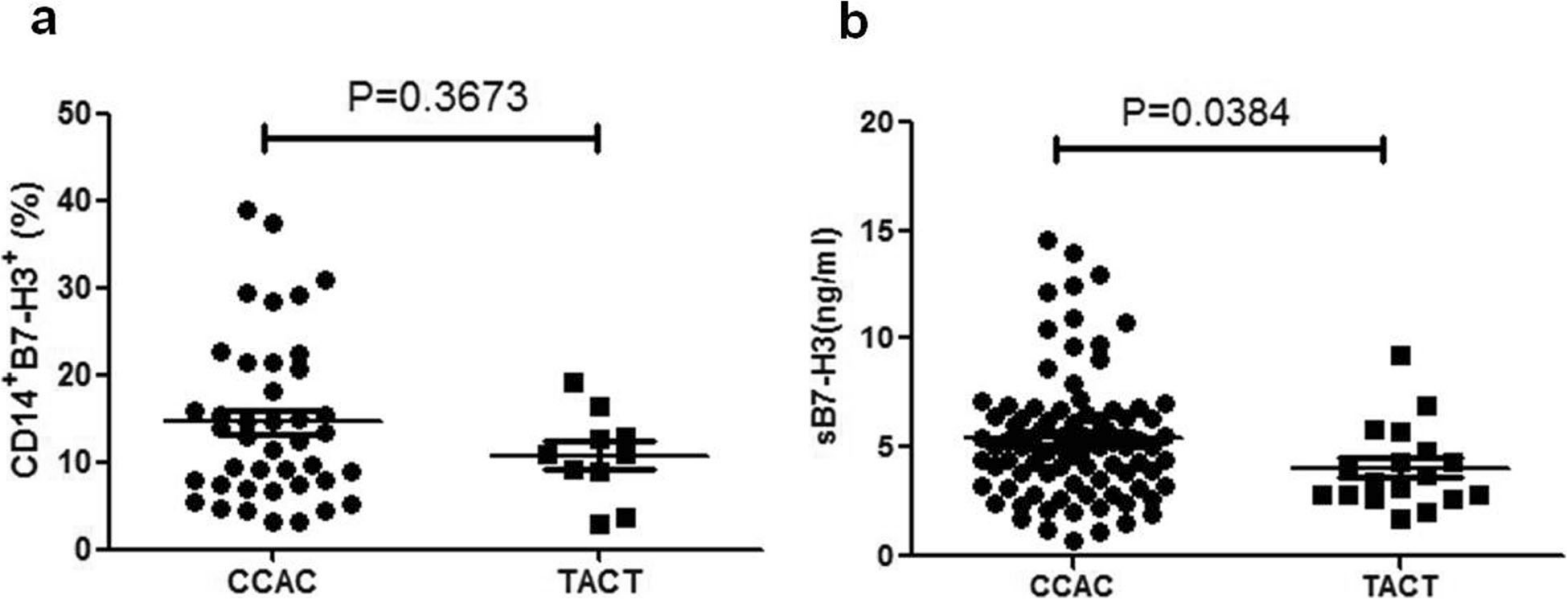
Association of the C-C-A-C gene and T-A-C-T gene polymorphisms of the B7-H3 gene with different isoform of B7-H3. The T1D patients were divided into two groups according to their C-C-A-C or T-A-C-T haplotype. The levels of membrane form of B7-H3 (**a**) and soluble form of B7-H3(**b**) are shown

We previously reported that the soluble form of B7-H3 was increased in T1D patients, and showed that titers of sB7-H3 were correlated with serum levels of Cr, BUN, ACR, HDL and gender [15]. In this study, the average concentration of sB7-H3 was (5.111 ± 2.821) ng/ml and (3.321 ± 1.729) ng/ml in T1D patients and healthy controls (*P* < 0.0001) respectively (Fig. 1b). The expression of membrane B7-H3 had no significant difference between the T1D patients carrying the different haplotypes (Fig. 2a). Concurrently we measured the concentration of sB7-H3 in the serum of T1D patients carrying different haplotypes and found that the T1D patients with T-A-C-T haplotype had lower concentrations of sB7-H3 when compared with the C-C-A-C haplotype (*P* = 0.0384; Fig. 2b).

## Discussion

Immune system and disease development are closely related and immune response can protect the body against different types of diseases. Therefore, it is critical to understand the co-stimulatory and co-inhibitory signals on multiple immune cell types in T1D. In this study, our research demonstrated that the expression of mB7-H3 on monocytes was significantly higher than healthy controls. The B7-H3-T-A-C-T polymorphism variant was associated with a decreased risk of T1D and furthermore was correlated with the secretion of sB7-H3 in serum.

The B7 family, including the CD28/B7 axis, ICOS/ICOSL pathway, receptors like TIM3 and B7-H4, is important for the regulation of immune responses mediated by antigen-specific T cells and also has a significant impact on T1D [21–25]. As an important member of the B7 family, the role of B7-H3 (CD276) in T1D remains unclear. B7-H3 was identified in 2001 as a cell surface molecule in the B7 immunoglobulin superfamily, which played an important role in the initiation and termination of immune cell responses as well as cancer development [8]. However, its receptor has not been identified.

One study found the involvement of B7-H3 in the innate immune monocyte/macrophage-mediated inflammatory response and confirmed that B7-H3 was associated with human sepsis and could augment the inflammatory responses [26]. In line with the study above, our previous experiments showed that B7-H3 was not expressed on T cells [18]. In addition to the investigation of the expression of mB7-H3 on CD14^+^ monocytes, we also observed increased levels of sB7-H3 in T1D patients when compared with healthy controls. A variety of co-stimulatory molecules exist both in soluble and membrane forms. Soluble co-stimulatory molecules could be produced by proteolytic cleavage and/or splicing of mRNA [27, 28]. Therefore, soluble form of B7-H3 in T1D may due to the cleavage of its membrane form. High mB7-H3 levels on monocytes along with the upregulation of sB7-H3 in patients suggested its co-stimulatory function, which stimulated the self-tolerance breakdown and thus led to the autoimmunity. Our results revealed that B7-H3 played a stimulatory role in T1D. The basic mechanism of controlling and regulating its production in different types of cells remains to be elucidated.

Since monocytes are the key producers of inflammatory factors and several studies also demonstrated that the intermediate CD14^+^ monocytes produced TNF-α in abundance and monocytes may play an essential role in promoting the inflammatory response in T1D [29, 30]. Previous studies have shown the association of B7-H3 with inflammatory reactions [26]. B7-H3 could amplify LPS, NF-κB p65 and MAPK p38 signals and make them participate in monocyte/macrophage-mediated inflammatory responses [26, 31]. B7-H3 also participated in the progression of asthma and augmented the inflammatory response independent of the Toll-like receptor 2 (TLR2) pathways [32]. Consistent with the above results, our study indicated the proinflammatory role of B7-H3 in the progression of T1D.

In addition, we studied the relationship between clinical features and levels of mB7-H3 in T1D patients. The expression of mB7-H3 was significantly correlated with the secretion of IFN-γ and TNF-α. We accorded these results with initial studies where B7-H3 synergistically promoted the secretion of cytokine IFN-γ and moderately upregulated the TNF-α. Significant correlation of the expression B7-H3 in T1D and the remaining clinical features could be explained with the co-stimulatory effect. Compared with the above results, our former study reported that sB7-H3 and renal function (Cr, BUN, ACR) was positively correlated and assumed that the soluble form may engage in the progression of diabetic nephropathy [15]. With all these results discussed above, we hypothesized that B7-H3 might act as a co-stimulator of innate immunity, which led to pathological damage by activating inflammatory response as well as enhancing T cell- mediated immune response. The upregulation of B7-H3 in the pathogenesis of T1D resulted in the pancreatic islet suffering autoimmune destructions. Previously, our group reported that patients suffering from rheumatoid arthritis (RA) had a higher frequency of T-A-T-C haplotype of B7-H3, and confirmed that it was associated with RA risk and affected the release of the soluble form from the cell surface [17]. Herein, B7-H3-T-A-C-T was observed to associate with the protective role of T1D, and similarly, with soluble B7-H3 expression.

Despite the above findings, our research has some shortcomings due to the lack of human samples. In addition, we could not speculate possible answers when having less samples. More detailed study is recommended with larger samples to investigate the existence of a haplotype-dependent distribution pattern for B7-H3 expression in Chinese individuals and T1D patients.

## Conclusions

In this study, we found a significant upregulation of mB7-H3 on monocytes and its correlation with several clinical features including ALT, fast C-peptide 120 min, HbAlc, IFN-γ, IL-6 and TNF-α in T1D patients. Moreover, the B7-H3 haplotype T-A-C-T was less frequently observed in T1D patients and was correlated with the decreased levels of soluble form but not the membrane form of B7-H3. Therefore, the B7-H3-T-A-C-T polymorphism variant was associated with less risk of T1D as well as the release of sB7-H3. The findings of this study indicated that B7-H3 might act as a potential biomarker related to the pathogenesis of T1D.

## Supplementary information

**Additional file 1: Fig. S1.** Expression of mB7-H3 in HC and T1D patients. Flow cytometry detection of B7-H3 on CD14^+^ monocytes in PB samples from healthy controls and T1D patients.

**Additional file 2: Fig. S2**. Polymorphisms of B7-H3 gene in this study. Raw data of electropherograms showed the mutant types of SNPs: rs7173448 (a), rs7173476(b), 1359 A → C (c), rs145827704 (d).

**Additional file 3: Supplementary Table 1.** The association analysis of SNP1 with T1D (adjusted by sex) by using logistic regression.

**Additional file 4: Fig. S3**. Linkage disequilibrium analysis in B7-H3 gene. D is the deviation between the expected haplotype frequency (under the assumption of no association) and the observed frequency; D’ is a proportion of the maximum value of D, which is scaled in [− 1,1] range; R is the correlation coefficient between alleles.

**Additional file 5: Fig. S4**. Expression of mB7-H3 in HC and T1D patients. Bars show the mean and SD of mB7-H3 mean fluorescence intensity (MFI).

## Data Availability

The datasets generated and/or analyzed during the current study are available from the corresponding author on reasonable request.

## Abbreviations

T1D: Type 1 Diabetes
SNP: single nucleotide polymorphisms
PCR: polymerase chain reaction
mB7-H3: membrane form of B7-H3
sB7-H3: soluble form of B7-H3
MFI: mean fluorescence intensity
Cr: creatinine
BUN: blood urea nitrogen
UA: uric acid
ALT: alanine aminotransferase
AST: aspartate transferase
TC: cholesterol
TG: triglycerides
LDL: low-density lipoprotein
HDL: high-density lipoprotein
IAA: insulin autoantibody
