# Supplementary figures and images for: Evaluation of the role of B7-H3 haplotype in association with impaired B7-H3 expression and protection against type 1 diabetes in Chinese Han population
Source: BMC Endocr Disord. 2020 Aug 12;20:123. doi: 10.1186/s12902-020-00592-7 (PMC7425597; doi:10.1186/s12902-020-00592-7)

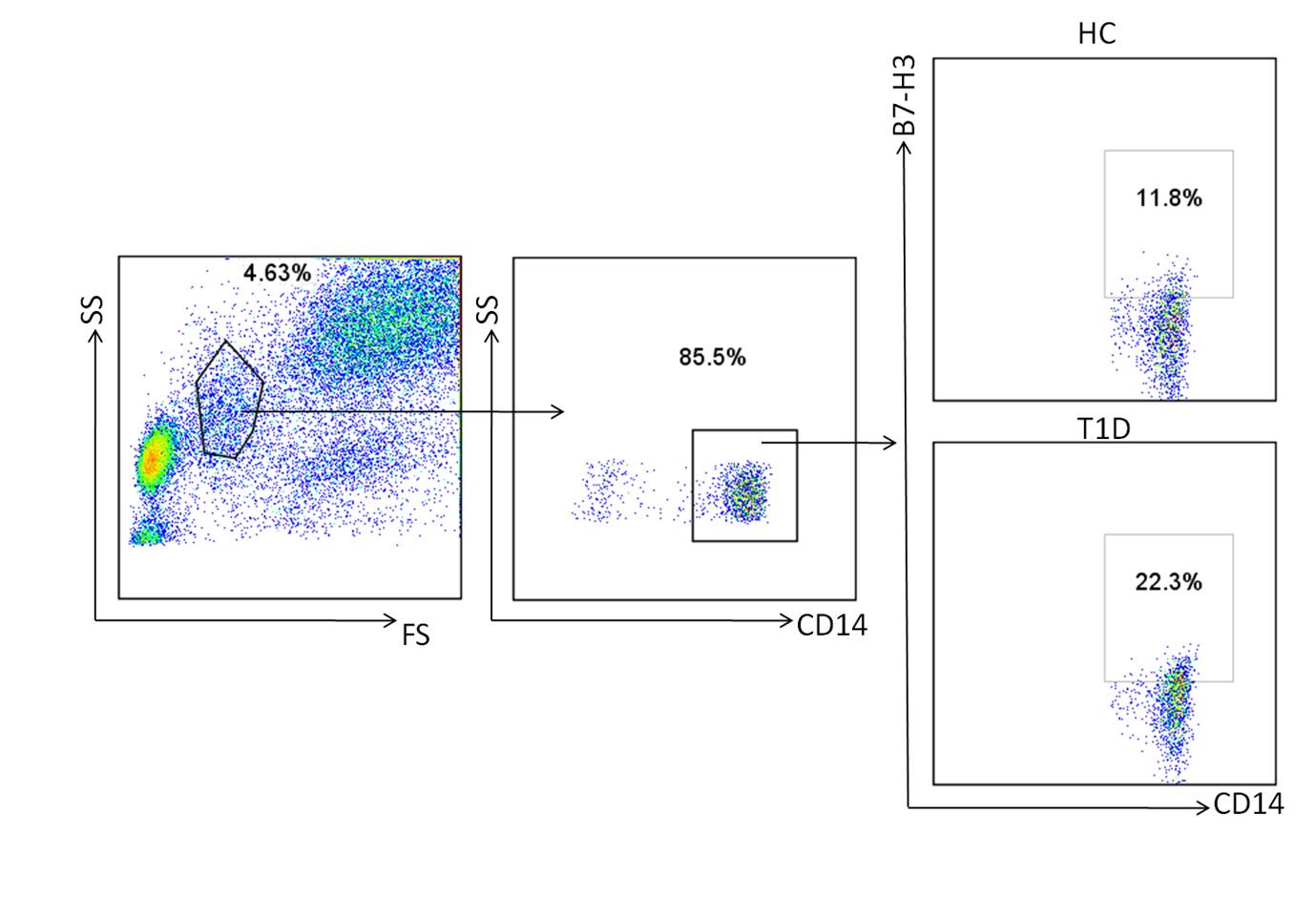

Supplement: Supplementary file 1 — Additional file 1: Fig. S1. Expression of mB7-H3 in HC and T1D patients. Flow cytometry detection of B7-H3 on CD14+ monocytes in PB samples from healthy controls and T1D patients. [file 12902_2020_592_MOESM1_ESM.jpg]

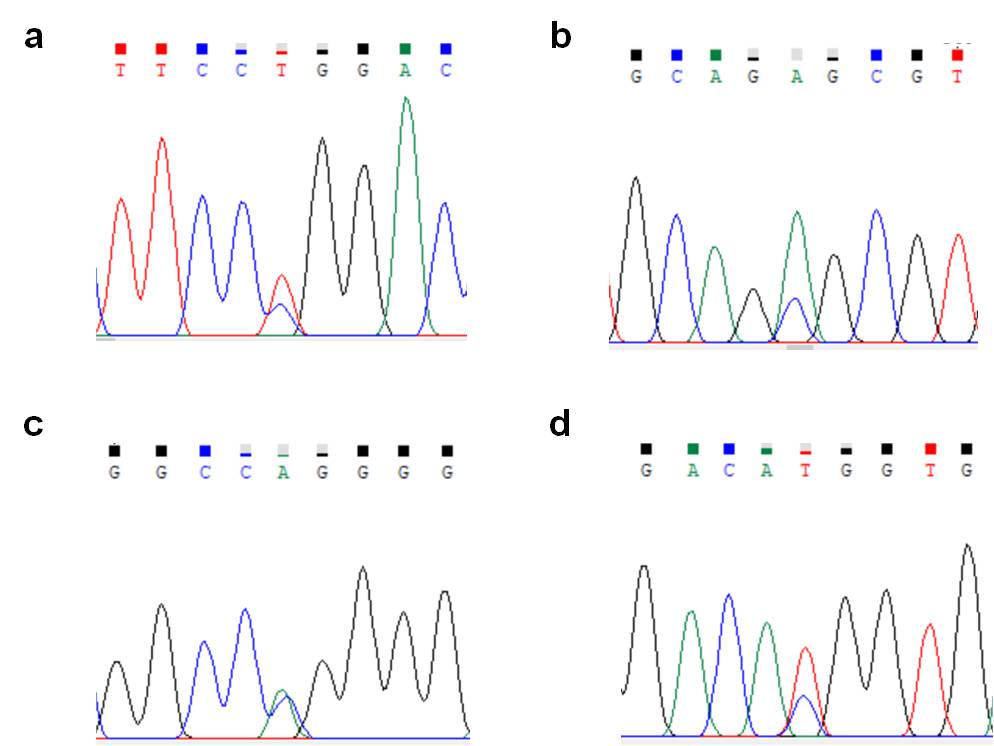

Supplement: Supplementary file 2 — Additional file 2: Fig. S2. Polymorphisms of B7-H3 gene in this study. Raw data of electropherograms showed the mutant types of SNPs: rs7173448 (a), rs7173476(b), 1359 A → C (c), rs145827704 (d). [file 12902_2020_592_MOESM2_ESM.jpg]

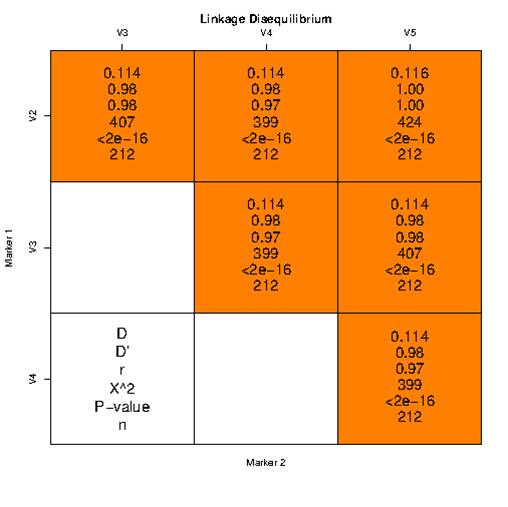

Supplement: Supplementary file 4 — Additional file 4: Fig. S3. Linkage disequilibrium analysis in B7-H3 gene. D is the deviation between the expected haplotype frequency (under the assumption of no association) and the observed frequency; D’ is a proportion of the maximum value of D, which is scaled in [− 1,1] range; R is the correlation coefficient between alleles. [file 12902_2020_592_MOESM4_ESM.jpg]

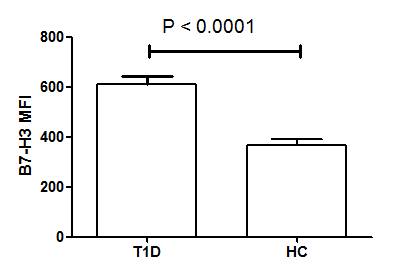

Supplement: Supplementary file 5 — Additional file 5: Fig. S4. Expression of mB7-H3 in HC and T1D patients. Bars show the mean and SD of mB7-H3 mean fluorescence intensity (MFI). [file 12902_2020_592_MOESM5_ESM.jpg]
