# Supplementary material for: Evaluation of the role of B7-H3 haplotype in association with impaired B7-H3 expression and protection against type 1 diabetes in Chinese Han population
Source: BMC Endocr Disord. 2020 Aug 12;20:123. doi: 10.1186/s12902-020-00592-7 (PMC7425597; doi:10.1186/s12902-020-00592-7)
Supplement: Supplementary file 3 — Additional file 3: Supplementary Table 1. The association analysis of SNP1 with T1D (adjusted by sex) by using logistic regression. [file 12902_2020_592_MOESM3_ESM.doc]

| **Supplementary Table 1** The association analysis of SNP1 with T1D(adjusted by sex) by using logistic regression | | | | | | | | | | | |
| --- | --- | --- | --- | --- | --- | --- | --- | --- | --- | --- | --- |
| Model | Genotype | Cases (%) | | Controls (%) | OR (95% CI) | | *P*-value | AIC | BIC | |  |
| Codominant | C/C | 84.8 | 66.4 | | 1 | 0.0064 | | 289.8 | | 299.8 | |
|  | C/T | 13.3 | 30.8 | | 0.34(0.17-0.68) |
|  | T/T | 1.9 | 2.8 | | 0.53(0.09-3.27) |
| Dominant | C/C | 84.8 | 66.4 | | 1 | 0.0016 | | 288 | | 294.7 | |
|  | C/T-T/T | 15.2 | 33.6 | | 0.35 (0.18-0.69) |
| Recessive | C/C-C/T | 98.1 | 97.2 | | 1 | 0.67 | | 297.7 | | 304.4 | |
|  | T/T | 1.9 | 2.8 | | 0.670(0.11-4.11) |
| Overdominant | C/C-T/T | 86.7 | 69.2 | | 1 | 0.0019 | | 288.2 | | 294.9 | |
|  | C/T | 13.3 | 30.8 | | 0.34(0.17-0.69) |
| Log-additive | --- | --- | --- | | 0.43(0.24-0.78) | 0.0038 | | 289.5 | | 296.2 | |
|  |  |  |  | |  |  | |  | |  | |

OR, odds ratio; 95% CI, 95% confidence intervals; AIC, Akaike’s information criterion; BIC,Bayesian information criterion
